# Oct4 Methylation-Mediated Silencing As an Epigenetic Barrier Preventing Müller Glia Dedifferentiation in a Murine Model of Retinal Injury

**DOI:** 10.3389/fnins.2016.00523

**Published:** 2016-11-15

**Authors:** Luis I. Reyes-Aguirre, Monica Lamas

**Affiliations:** Departamento de Farmacobiología, Centro de Investigación y de Estudios Avanzados-Sede SurMéxico, Mexico

**Keywords:** Müller glia, DNA methylation, retinal progenitors

## Abstract

Müller glia (MG) is the most abundant glial type in the vertebrate retina. Among its many functions, it is capable of responding to injury by dedifferentiating, proliferating, and differentiating into every cell types lost to damage. This regenerative ability is notoriously absent in mammals. We have previously reported that cultured mammalian MG undergoes a partial dedifferentiation, but fails to fully acquire a progenitor phenotype and differentiate into neurons. This might be explained by a mnemonic mechanism comprised by epigenetic traits, such as DNA methylation. To achieve a better understanding of this epigenetic memory, we studied the expression of pluripotency-associated genes, such as *Oct4, Nanog*, and *Lin28*, which have been reported as necessary for regeneration in fish, at early times after NMDA-induced retinal injury in a mouse experimental model. We found that although Oct4 is expressed rapidly after damage (4 hpi), it is silenced at 24 hpi. This correlates with a significant decrease in the DNA methyltransferase *Dnmt3b* expression, which returns to basal levels at 24 hpi. By MS-PCR, we observed a decrease in *Oct4* methylation levels at 4 and 12 hpi, before returning to a fully methylated state at 24 hpi. To demonstrate that these changes are restricted to MG, we separated these cells using a GLAST antibody coupled with magnetic beads. Finally, intravitreous administration of the DNA-methyltransferase inhibitor SGI-1027 induced *Oct4* expression at 24 hpi in MG. Our results suggest that mammalian MG injury-induced dedifferentiation could be restricted by DNA methylation, which rapidly silences *Oct4* expression, preventing multipotency acquisition.

**Key Points**

A silencing event upon the pluripotency marker Oct4 expression occurs in mice Müller glia after retinal damage.

This event correlates with decreased DNA methyltransferase expression and Oct4 methylation profile.

## Introduction

Müller glia (MG) is responsible for the maintenance of retinal homeostasis and neural nourishment (Pfeiffer-Guglielmi et al., 2005), protection against oxidative stress (Schütte and Werner, 1998), maintenance of the blood-retinal barrier (BRB; Bringmann et al., 2006), growth factor release (Eichler et al., 2001), and even light guidance to the photoreceptor layer (Agte et al., 2011).

MG is remarkably capable of regenerating damaged neurons, albeit in a very restricted number of vertebrates, particularly teleost fish, by dedifferentiating and acquiring a progenitor phenotype. This is revealed by a drastic change in MG's gene expression program, as they down-regulate and eventually lose the expression of its specific markers cellular retinaldehyde-binding protein (CRALBP) and glutamine synthase (GS), while up-regulating progenitor-associated markers such as *ascl1a, chx10*, and *six3*, and genes usually expressed by pluripotent cells, like *oct4, lin28, sox2, klf4*, and c-*myc* (Raymond et al., 2006; Bernardos et al., 2007; Fischer and Bongini, 2010; Ramachandran et al., 2010). These pluripotency-associated markers are usually present in embryonic stem cells (ESCs) and are often regarded as fundamental for the generation of iPSCs (Takahashi and Yamanaka, 2006; Hochedlinger and Plath, 2009). Notably, *Oct4* has been described as both necessary and sufficient for direct induction of multipotency in adult neural stem cells (NSCs; Kim et al., 2009). The dedifferentiation stage allows MG to proliferate and then differentiate into neurons and completely restore visual function.

In mammals, MG responds to damage by becoming reactive and hypertrophic, in a process known as reactive gliosis which involves the up-regulation of glial fibrillary acidic protein (GFAP) and GS, as well as uncontrolled proliferation (Fawcett and Asher, 1999). Far from restoring visual function, this ultimately contributes to neurodegeneration and loss of visual function. While naturally occurring dedifferentiation-mediated regeneration is missing in these organisms, several research groups have demonstrated that, under specific conditions, mammalian MG has the basic machinery needed to initiate such a process, acquiring a progenitor phenotype, and even differentiating to other cellular types, although with varying grades of success (Ooto et al., 2004; Karl et al., 2008; Abrahan et al., 2009; Stutz et al., 2014).

We have previously reported that low, sub-toxic concentrations of glutamate are enough to trigger the expression of some progenitor-associated markers (*Lin28* and *Chx10*, and nestin) in most Müller cells in culture, at very short times after treatment (1 and 4 h). However, only 2% of the allegedly dedifferentiated cells expressed early neuronal markers after applying proven differentiation protocols, and none of them fully acquired a neuronal phenotype. Also, cells in culture failed to down-regulate the MG-specific markers CRALBP and GS (Reyes-Aguirre et al., 2013). We reasoned that this partial dedifferentiation could be explained by a mnemonic mechanism restricting treated cells to a glial phenotype. Such a mechanism can be seamlessly associated with epigenetics, since the transcriptional machinery responsible for changes in gene expression is controlled by epigenetic traits.

DNA methylation is the most stable among epigenetic mechanisms, and is considered the main device of epigenetic memory. It involves the covalent bonding of methyl groups to the C-5 position of the cytosine ring within a DNA sequence, mediated by DNA methyltransferases (DNMTs; Jin et al., 2011). This modification correlates negatively with gene transcription and it is consistently observed in high levels throughout the genome of somatic cells. Three major DNA methyltransferases have been identified in mammals: DNMT1, classically considered a maintenance methyltransferase (Rhee et al., 2012) and DNMT3a and DNMT3b, which can initiate methylation of previously unmethylated DNA (Chen et al., 2003). This modification is reversed by DNA demethylation, which, in the absence of an specific demethylase, potentially involves several different proteins, including TET, GADD45, and deaminases (Rai et al., 2008, 2010).

In this study, we identified changes in the expression of pluripotency-associated genes, previously reported in zebrafish, in a mammalian retinal injury model, at early time points after damage. We suggest a possible correlation between changes in these genes and in those related to DNA methylation and demethylation, whose blockage should enhance MG dedifferentiation.

## Materials and methods

### Animal subjects

Adult (6–10 weeks) C57BL/6J mice were used for all lesion experiments and subsequent analysis. Animals were housed under standard conditions with access to water and food *ad libitum*. All procedures were performed under protocols approved by the internal animal care committee (CICUAL–CINVESTAV), and following the guidelines of the Association for Research in Vision and Ophthalmology (ARVO) Statement for the Use of Animals in Ophthalmic and Vision Research.

### NMDA intravitreal injection and eye extraction

Mice were anesthetised with pentobarbital (0.1 mg per 10 g body weight) by an i.p. injection. Tetracaine topical anesthetic was applied to the eyes before the intravitreal injection, as well as a tropicamide/phenylephrine ophthalmic solution. The animals were then placed on a head mount, and a 30G needle was carefully inserted at the upper temporal ora serrata in the left eye, delivering 250 mM N-methyl-D-aspartate (NMDA) (Sigma) into the vitreous humor. The right eye remained intact as a control. After the injection, mice were returned to their home cages for recovery, with food and water *ad libitum*. Eyes were enucleated at 4, 12, 18, and 24 h post injury (hpi), and prepared for further analysis.

### Immunohistochemistry

After enucleation, eyes were fixed with a 4% paraformaldehyde (PFA) solution for 1 h at 4°C. Following fixation, the anterior region was removed and the posterior part cryopreserved in a 30% sucrose solution overnight at 4°C. Then, eyes were embedded in OCT compound (Tissue-Tek, Sakura Finetek, USA) and frozen for cryo-sectioning with a microtome (Leica), obtaining 10 μm retinal sections placed on coated slides (Biocare Medical, USA), which were air-dried for 1 h at room temperature (RT) and then hydrated for 30 min with phosphate buffered saline (PBS). The slides were placed in a wet chamber and blocked with a 0.3% Triton X-100 solution containing 1% bovine serum albumin (BSA) and 5% goat serum for 1 h at RT. Primary antibodies against nestin (1:200; Millipore, Billerica, MA, USA, MAB353), and glutamine synthase (GS) (1:100; Abcam, AB16802), were diluted in PBS and allowed to bind to the slides overnight at 4°C. After several rounds of washing, the slides were incubated with the secondary antibodies Alexa 488 anti-mouse (1:500; Molecular Probes, Eugene, OR, USA) and/or Alexa 568 anti-rabbit (1:500; Molecular Probes, Eugene, OR, USA), diluted in PBS with 4′,6-diamidino-2-phenylindole (DAPI; 1:15000; Sigma) for 1 h and 30 min at RT. The slides were then mounted with DABCO, and viewed on an epifluorescence microscope Zeiss Axiovert 40 CFL, coupled with a digital camera Carl Zeiss Axiocam MRm, with AxioVision Rel.4.8 software.

### Terminal deoxynucleotidyl transferase dUTP nick-end labeling (TUNEL) assay

Cell death was analyzed in slides from eyes enucleated 24 hpi, using the *in situ* Cell Death Detection Kit, Fluorescein (Roche Diagnostics, GmbH, Mannheim, Germany), according to manufacturer's instructions. As a positive control, some slides were incubated with 200 U/ml DNase I (Sigma) for 10 min at RT.

### RNA extraction, RT-PCR, and qPCR

Total RNA was isolated from enucleated eyes using Trizol (Invitrogen, CA, USA), from which complementary DNA was synthesized using Oligo dT and Superscript-II reverse transcriptase (Invitrogen). Specific cDNAs were amplified by PCR over 30–40 cycles, using *Taq* polymerase (Fermentas) and gene-specific primers (Table 1), under the following conditions: denaturation at 95°C for 30 s, annealing at 50–60°C (this temperature was changed according to the primers' Tm) for 15 s, and extension at 72°C for 30 s. The PCR products were resolved on 1.5% agarose gels containing 25 ng/ml ethidium bromide, and visualized in an UV EpiChem^3^ Darkroom transilluminator. Images were captured using the LabWorks 4.5 software (BioImaging Systems, UVP, Upland, CA, USA). Embryonic (E10) cDNA was used as a positive control, while non-template samples were negative controls.

**Table 1:** **Primers used for RT-PCR and qPCR analysis**.

**Gene**		**Sequence**
Oct4	Forward	TCTTTCCACCAGGCCCCCGGCTC
	Reverse	TGCGGGCGGACATGGGGGAGATCC
Nanog	Forward	GACTGAGATATGGCTTGCTC
	Reverse	CTTTCTGAGGGATAGGGTCT
Klf4	Forward	GGCGAGTCTGACATGGCTG
	Reverse	GCTGGACGCAGTGTCTTCTC
Pax6	Forward	CGGAGGGAGTAAGCCAAGA
	Reverse	AAGGGCACTCCCGTTTATACT
Lin28	Forward	AGGCGGTGGAGTTCACCTTTAAGA
	Reverse	AGCTTGCATTCCTTGGCATGATGG
Sox2	Forward	TAGAGCTAGACTCCGGGCGATGA
	Reverse	TTGCCTTAAACAAGACCACGAAA
C-myc	Forward	TGACCTAACTCGAGGAGGAGCTGGAATC
	Reverse	AAGTTTGAGGCAGTTAAAATTATGGCTG
DNMT1	Forward	CCTAGTTCCGTGGCTACGAGGAGAA
	Reverse	TCTCTCTCCTCTGCAGCCGACTCA
DNMT3a	Forward	GCCGAATTGTGTCTTGGTGGATGACA
	Reverse	CCTGGTGGAATGCACTGCAGAAGGA
DNMT3b	Forward	TGGGTACAGTGGTTTGGTGA
	Reverse	GCCCTTGTTGTTGGTGACTT
Gadd45a	Forward	CCTGCACTGTGTGCTGGTGA
	Reverse	CCACTGATCCATGTAGCGACTTTC
Gadd45b	Forward	CCTGGCCATAGACGAAGAAG
	Reverse	AGCCTCTGCATGCCTGATAC
Tet1	Forward	GAGCCTGTTCCTCGATGTGG
	Reverse	CAAACCCACCTGAGGCTGTT
Tet2	Forward	GCCATTCTCAGGAGTCACTGC
	Reverse	ACTTCTCGATTGTCTTCTCTATTGAGG
Tet3	Forward	GGTCACAGCCTGCATGGACT
	Reverse	AGCGATTGTCTTCCTTGGTCAG
GAPDH	Forward	ACTGGCATGGCCTTCCGTGTTCCTA
	Reverse	TCAGTGTAGCCCAAGATGCCCTTC

Quantitative PCR reactions were performed using KAPA SYBR FAST (Kappa BioSystems, Wilmington, MA, USA) master mix on the PikoReal Real-time PCR System (Thermo Scientific), for 35 cycles as following: denaturation at 94°C for 10 s, annealing at 50–60°C for 30 s, and extension at 72°C for 30 s. Reactions for each primer were performed in triplicate. The ΔΔCT method was used to determine mRNA levels in control and injured retinas. All data were normalized to GAPDH mRNA expression levels.

### Bisulphite conversion and methylation-specific PCR (MSPCR)

After eye enucleation and retinal extraction, genomic DNA was extracted with the EpiTect Fast DNA Bisulfite Kit (Qiagen, Hilden, Germany), and then treated for bisulphite conversion and purified according to manufacturer's instructions. MSPCR was performed using the EpiTect MSP kit (Qiagen, Hilden, Germany) master mix and previously reported specific primers for the methylated and unmethylated forms of *Oct4* (Wang et al., 2013; Table 2). DNA was amplified for 40 cycles, which comprised a denaturation step at 94°C for 15 s, annealing at 50°C for 30 s, and extension at 72°C for 30 s. The resulting products were resolved on 1.5% agarose gels containing 25 ng/ml ethidium bromide, and visualized in an UV EpiChem^3^ Darkroom transilluminator. Images were captured using the LabWorks 4.5 software (BioImaging Systems, UVP, Upland, CA, USA). Samples treated with CpG methyltransferase M.SssI (New England BioLabs, Ipswich, MA, USA) were used as positive methylation controls.

**Table 2:** **Primers for MSPCR for Oct4**.

**Gene**		**Sequence**
Oct4 M	Forward	GGTTTTAGAAATAATTGGTATACG
	Reverse	CTATTAACACTACACCCTCTCGAC
Oct4 U	Forward	TGGTTTTAGAAATAATTGGTATATGA
	Reverse	CCTATTAACACTACACCCTCTCAAC

*M, methylated allele; U, unmethylated allele*.

### High resolution melting (HRM) analysis

DNA was amplified for HRM analysis on the PikoReal Real-time PCR System (Thermo Scientific), using a Luminaris Color HRM (Thermo Scientific) master mix, in a volume of 20 μl, containing 20 ng of DNA. Thermal cycling conditions were as follows: denaturation at 95°C for 10 s, annealing at 50°C for 30 s, and extension at 72°C for 30 s, for 40 cycles. Samples were then melted from 40 to 99°C, with a melting rate of 0.2°C/s. All HRM data were collected and analyzed by the dedicated PikoReal software (Thermo Scientific, version 2.2). Normalization of HRM curves was performed by the same software, as well as calculation of closest standard call (value attributed to each sample with respect to a methylation standard). All analysis were performed in triplicate. Genomic DNA from intact retinas, treated with CpG methyltransferase M.SssI (New England BioLabs, Ipswich, MA, USA) was used as methylation standard.

### Magnetic-activated cell sorting (MACS)

As previously reported (Eberle et al., 2014), isolated retinas were dissociated in DMEM containing 0.5% trypsin (Sigma) for 20 min at 37°C. The enzymatic reaction was inhibited by transferring the tissue to DMEM containing 10% fetal calf serum. Additional mechanical dissociation was performed by triturating cells with a 1 ml plastic tip and then a fire-polished glass Pasteur pipette. Cells were resuspended in MACS buffer and incubated with a rabbit anti-mouse primary antibody against GLAST (1:200; Novus Biologicals, Littleton, CO, USA, NB100-1869) for 5 min at 4°C, washed in MACS buffer, and then centrifuged. The cell pellet was resuspended in MACS buffer and incubated with 0.1 ml of goat anti-rabbit IgG magnetic beads (Miltenyi Biotec, Bergisch Gladbach, Germany) for 15 min at 4°C. Magnetic separation was performed according to manufacturer's instructions on a QuadroMACS separator, using LS columns (Miltenyi Biotec, Bergisch Gladbach, Germany). Briefly, the cells were placed on a column fixed to the separator and flow through (GLAST-negative cells) was collected. Then, GLAST-positive fraction was eluted by loading 5 ml MACS buffer and immediately applying pressure with the supplied plunger. Cells from both fractions were then prepared for further analysis.

### SGI-1027 intravitreal injection

DNA methyltransferase inhibitor SGI-1027 (Sigma) was dissolved in 0.05% DMSO, and injected intravitreally following the same procedure as NMDA injection (10 μM in 2 μl), 24 h before retinal injury.

### Statistical analysis

All experiments were performed by triplicate and all data are expressed as mean ± SEM. Statistical significance of the differences was assessed by a one-way analysis of variance (ANOVA), followed by a Tukey test for *post-hoc* comparisons, or by Student's *t*-test.

## Results

### Expression of pluripotency-associated markers after retinal injury

**Figure 1:**
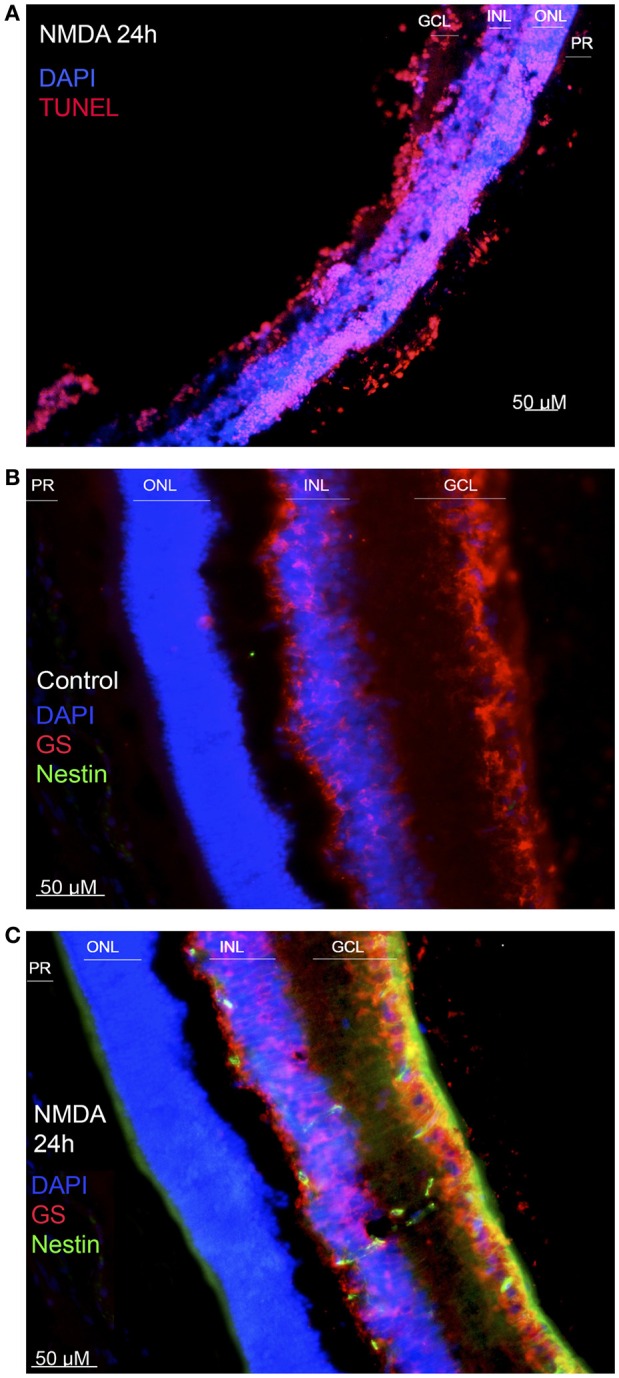
***Effect of NMDA injury*. (A)** TUNEL test performed on a damaged retina, 24 h after injury. Cell nuclei are shown in blue, and apoptotic cells in red. **(B)** Immunofluorescence against nestin and glutamine synthase (GS) on an intact retina. MG, positive to GS, is shown in red. No nestin-positive cells are detected. **(C)** Immunofluorescence against the same markers on a damaged retina, extracted 24 h after injury. Co-labeling of GS (red) and nestin (green) indicates that MG are expressing the progenitor-associated marker as a response to damage. (ONL, outer nuclear layer; INL, inner nuclear layer; GCL, ganglion cell layer; PR, photoreceptor layer). Calibration bar: 50 μm.

We demonstrated the excitotoxic effect of the NMDA injection by a TUNEL assay, comparing injured retinas (at 24 hpi) with positive controls. NMDA produced massive cellular death throughout all retinal layers (Figure 1A), as well as a general disruption of the laminar structure of the retina. MG response to damage was assessed by immunofluorescence against GS and nestin, a filamentous protein usually found in progenitor cells. Intact retinas (Figure 1B) lack nestin-immunopositive cells. After 24 hpi, co-labeling of nestin and GS is observable across the inner nuclear layer (INL), reflecting MG early dedifferentiation (Figure 1C).

**Figure 2:**
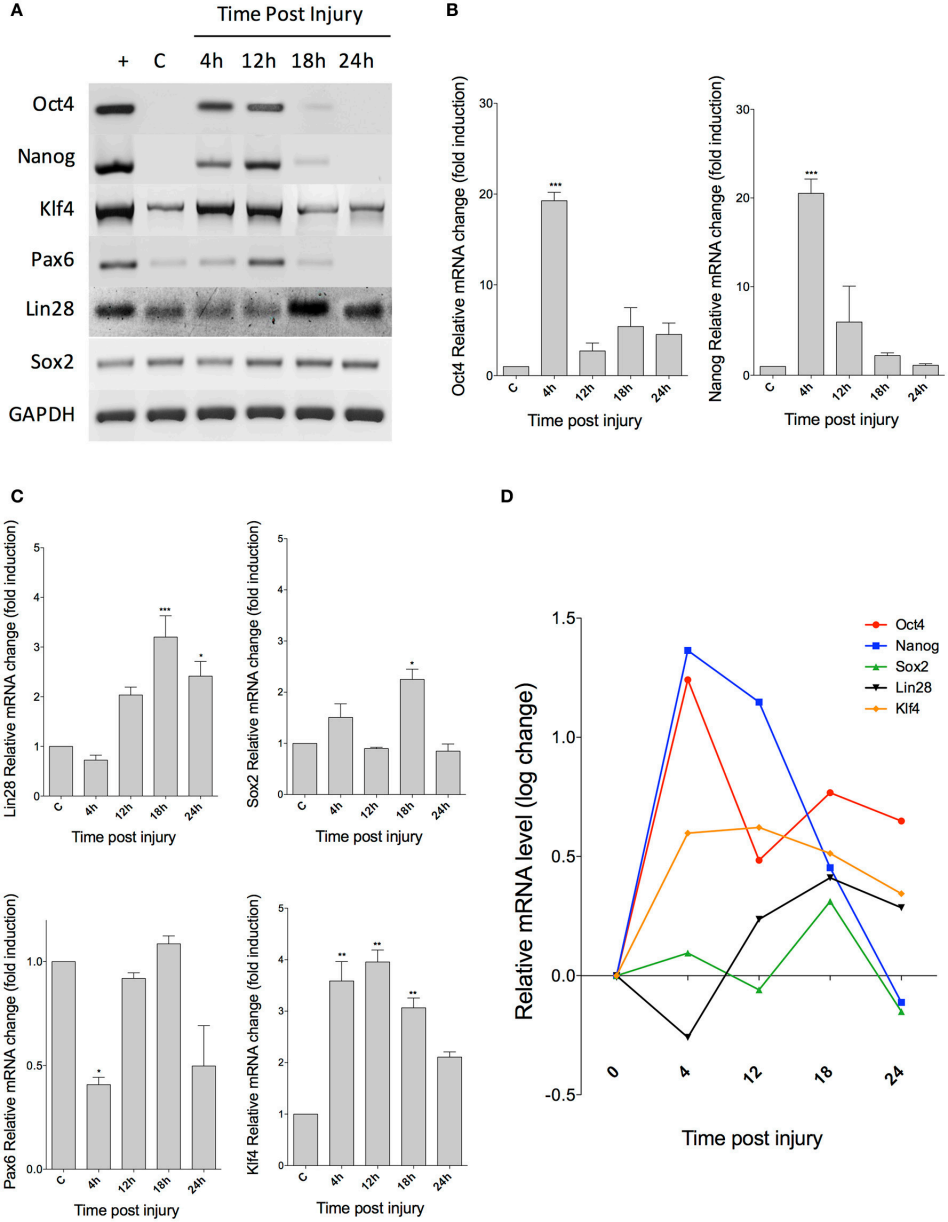
***Expression of pluripotency-associated markers in injured retina*. (A)** RT-PCR analysis reveals the changes in expression of several markers at the indicated times after injury. C, control; +, embryonic cDNA as positive control. **(B)** Real-time PCR quantification of Oct4 and Nanog expression levels **(C)** qPCR analysis for Lin28, Sox2, Pax6, and Klf4. **(D)** Change dynamics for all markers after a logarithmic transformation of data to allow a better visualization of the expression pattern for each gene. (ANOVA: ^***^p < 0.001; ^**^p < 0.01; ^*^p < 0.05).

To characterize the early molecular events during MG response to NMDA-induced injury in rodent retina *in vivo*, we analyzed the changes in expression of pluripotency-associated genes of retinas extracted at 4, 12, 18, and 24 hpi (Figure 2A) from a group of mice (*n* = 12, 3 per group). We found that *Oct4* and *Nanog*, both regarded as essential for pluripotency acquisition and maintenance, rapidly increase their expression after 4 and 12 hpi. However, this change is transient, and both genes become undetectable at 24 hpi. *Klf4* and *Pax6* also appear to have a transient expression, since both are substantially reduced after 18 hpi. In contrast, *Lin28* and *Sox2* maintain their expression throughout all time points. To validate these data, we performed qPCR analysis and determined the relative expression level of each gene. A one way ANOVA revealed that *Oct4* and *Nanog* reach an expression peak at 4 hpi (*p* < 0.001, when compared to controls) and then reduce their levels at 18 and 24 hpi (Figure 2B). In a similar fashion, *Klf4* exhibits a significant increase at 4, 12, and 18 hpi (*p* < 0.01) and then a reduction at 24 hpi. *Pax6* actually undergoes a slight decrease at 4 hpi (*p* < 0.05), before increasing its expression levels at 12 and 18 hpi (Figure 2C). In order to achieve a better visualization of the changes in mRNA levels for each gene, we compiled the data and performed a logarithmic transformation (Figure 2D). The resulting graph shows the expression dynamics of the evaluated pluripotency-associated genes.

### Expression of markers associated to DNA methylation and demethylation

**Figure 3:**
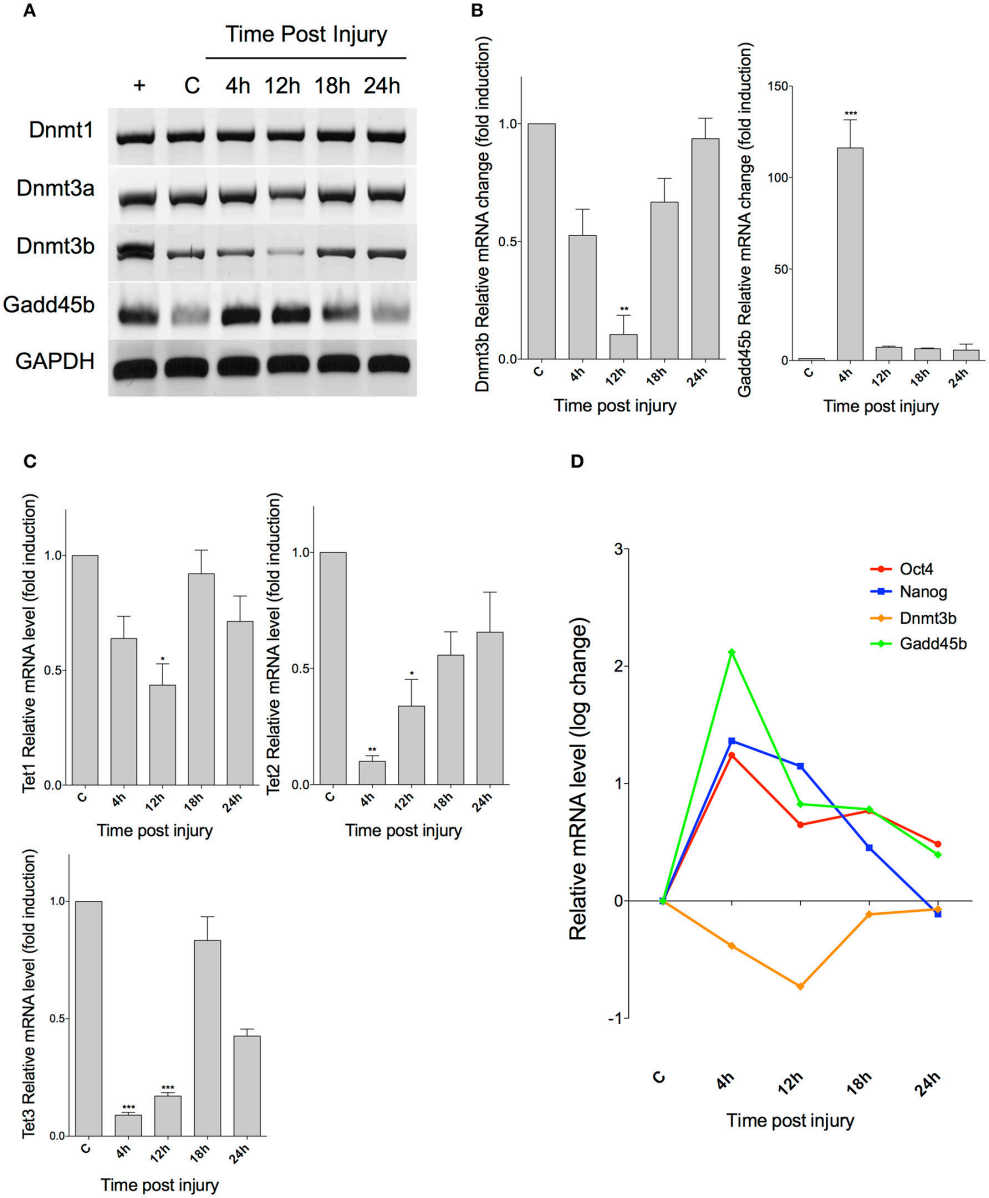
***Expression of markers associated to DNA methylation and demethylation after retinal injury in vivo*. (A)** RT-PCR analysis for genes encoding for DNA methyltransferases (Dnmt1, Dnmt3a, and Dnmt3b), and a Gadd45 protein, associated with repair-based DNA demethylation. C, control; +, embryonic cDNA used as positive control. **(B)** qPCR analysis of Dnmt3b and Gadd45b. **(C)** qPCR for DNA demethylation-associated Tet proteins. **(D)** Comparison between expression changes in pluripotency-associated genes and methylation and demethylation-associated markers (ANOVA; ^***^p < 0.001; ^**^p < 0.01; ^*^p < 0.05).

The transient expression of pluripotency-associated genes suggests the presence of a silencing mechanism acting on MG at 24 hpi. Since DNA methylation is considered the main device of epigenetic memory (Vierbuchen and Wernig, 2012), we chose to analyse the expression of genes encoding DNA methyltransferases and Gadd proteins, by RT-PCR (Figures 3A,B). As expected, we did not observe any change in the expression levels of the maintenance methyltransferase *Dnmt1*, but the expression of *Dnmt3a* and *3b*, which are involved in *de novo* methylation of DNA, is diminished at 4 and 12 hpi. Gadd proteins, associated with DNA demethylation, also exhibited changes in their expression profiles. Particularly, we found a significant increase of *Gadd45b* at 4 and 12 hpi. These mRNA is then drastically reduced at 18 and 24 hpi. Quantification of gene expression changes by qPCR demonstrated that *Dnmt3b* exhibits a biphasic, time-dependent decrease, which becomes statistically significant (by an ANOVA test) at 12 hpi (*p* < 0.01), before reverting to its basal levels. *Gadd45b* also shows a significant increase at 4 hpi (*p* < 0.001), and then returns to normal levels (Figure 3B). We also analyzed the expression of the genes that encode active DNA demethylation-associated Tet proteins (Figure 3C), and observed that, unexpectedly, the expression of *Tet1* is decreased at 12 hpi (*p* < 0.05); *Tet2* is decreased at 4 and 12 hpi (*p* < 0.01 and 0.05, respectively), and *Tet3* at 4, 12, and 24 hpi (*p* < 0.001, 0.001, and *p* < 0.01, respectively). We then performed a logarithmic transformation of data in order to achieve a better visualization, and compared the changes in the expression of *Dnmt3b* and *Gadd45b* with those of *Oct4* and *Nanog* (Figure 3D). Noteworthy, the expression peak of pluripotency genes correlates with a major decrease in *Dnmt3b* levels (at 4 and 12 hpi), and with a significant increase in *Gadd45b*.

### Oct4 methylation profile in injured retina

**Figure 4:**
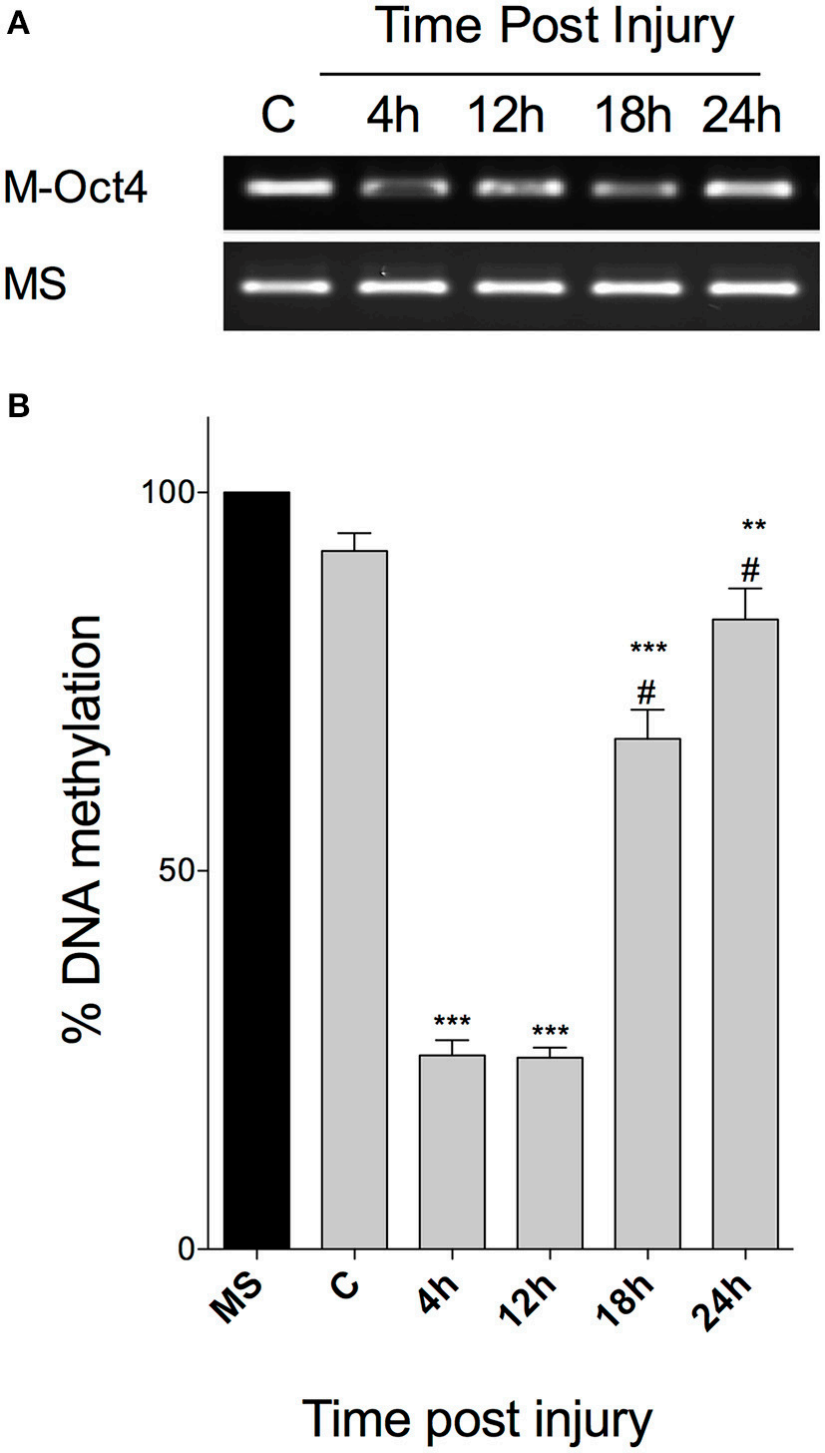
***Oct4 methylation profile after retinal injury in vivo*. (A)** MS-PCR of Oct4, with primers specific to its methylated form, resolved in an agarose gel. M-Oct4, Methylated form of Oct4; fully methylated DNA was used as an internal control. **(B)** Representation of the percentage of Oct4 methylation at different times after injury, calculated after HRM analysis, and compared to a methylation standard. (ANOVA; ^***^p < 0.001; ^**^p < 0.01, when compared to the fully methylated standard (MS); #*p* < 0.001, when compared with 4 and 12 hpi).

Given the apparent correlation between *Oct4, Dnmt3b*, and *Gadd45b*, we reasoned that *Oct4* DNA methylation profile might exhibit changes at different times post injury. To demonstrate this, we extracted genomic DNA from another group of mice (*n* = 12, 3 per group) and performed a methylation-specific PCR analysis, using previously tested primers, which recognize the methylated form of *Oct4* (Wang et al., 2013). We observed a decrease in methylated *Oct4* at 4 and 12 hpi, with an apparent return to basal levels at 24 hpi (Figure 4A). Assuming the limitations of MS-PCR, we also performed an HRM analysis, using several dilutions of DNA treated with CpG methyltransferase M.SssI as a methylation standard. By using an ANOVA test, we observed that NMDA injury induced a significant decrease in *Oct4* methylation after 4 and 12 hpi (down to 20% when compared to the fully methylated standard; *p* < 0.001; Figure 4B). Afterwards, DNA methylation tends to increase, up to 60% when compared with the standard (*p* < 0.001) at 18 hpi, and around 88% at 24 hpi (*p* < 0.01). These results indicate a tendency of Oct4 to return to its fully methylated state as damage proceeds.

### Damage response in mice is restricted to MG

**Figure 5:**
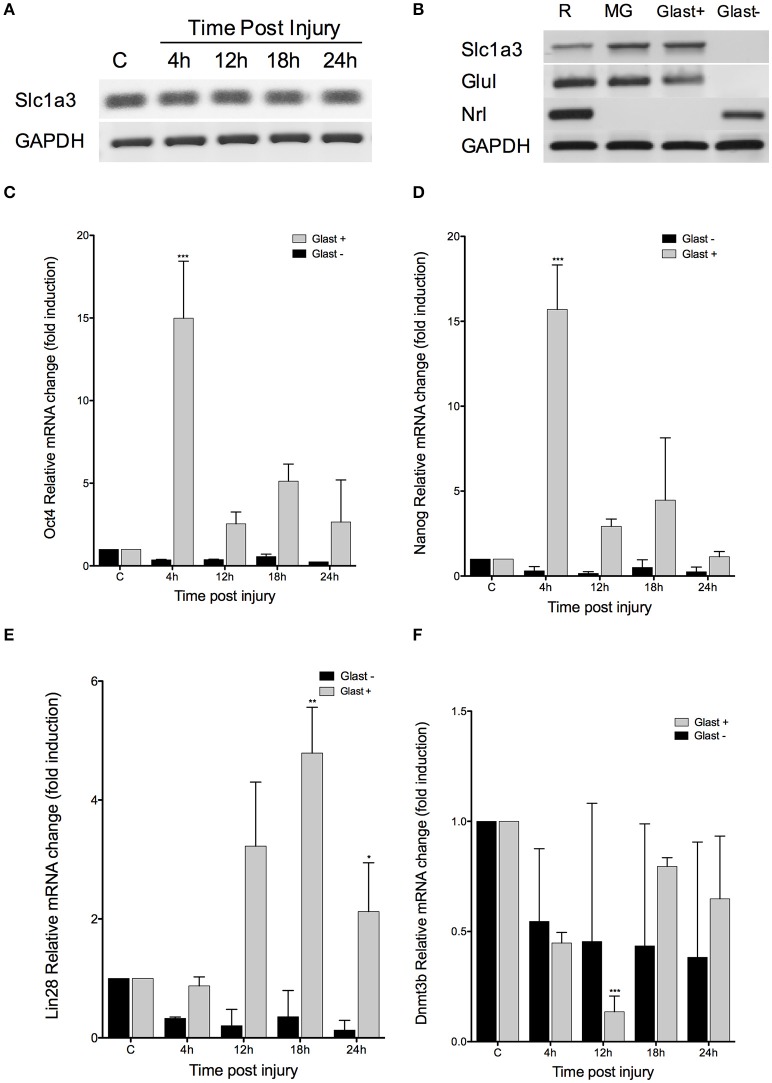
***Damage response is restricted to MG*. (A)** RT-PCR analysis for Slc1a3, the gene encoding GLAST, at the indicated times after injury. **(B)** RT-PCR for MG and a photoreceptor-specific marker (Nrl) in GLAST-positive and negative fractions, after MACS in intact retinas. **(C–F)** qPCR quantification of Oct4, Nanog, Lin28, and Dnmt3b expression levels at the indicated time after injury in MACS GLAST-positive and negative fraction; C, intact retina as control (Student's *t*-test; ^***^p < 0.001; ^**^p < 0.01; ^*^p < 0.05).

To evaluate whether the expression changes we observed are happening exclusively in MG, and not in other cell types in the retina, we separated Müller cells by magnetic associated cell sorting (MACS) after extracting retinas from 12 mice (3 per condition). This procedure depends on a reliable surface marker, and we chose the glutamate transporter GLAST, mainly expressed in radial glia in the central nervous system and specific to MG in the retina (Namekata et al., 2009). First, we confirmed that Slc1a3 (which encodes GLAST) maintains its expression levels *in vivo* after retinal injury (Figure 5A), making GLAST a suitable marker for MACS in our injury model. As a validation to our cell sorting results, we found that the GLAST-positive fraction shares the expression of both *Slc1a3* (GLAST) and *Glul* (GS) with cultured MG. In contrast, the GLAST-negative fraction expresses the photoreceptor marker *Nrl*, also found in a whole, intact retina sample (Figure 5B). We extracted total RNA from both GLAST-positive and negative fractions of injured retina for qPCR analysis, and found that changes in *Oct4* expression are restricted to the positive fraction, i.e., MG, closely resembling the pattern previously found in whole retina (Figure 5C). We obtained similar results after quantifying *Nanog* (Figure 5D), *Lin28* (Figure 5E), and *Dnmt3b* (Figure 5F). These genes are expressed exclusively in MG after retinal injury, and show no significant changes in GLAST-negative cells, after statistical analysis by a Student's *t*-test.

### DNA methylation blockage maintains Oct4 expression at 24 hpi

**Figure 6:**
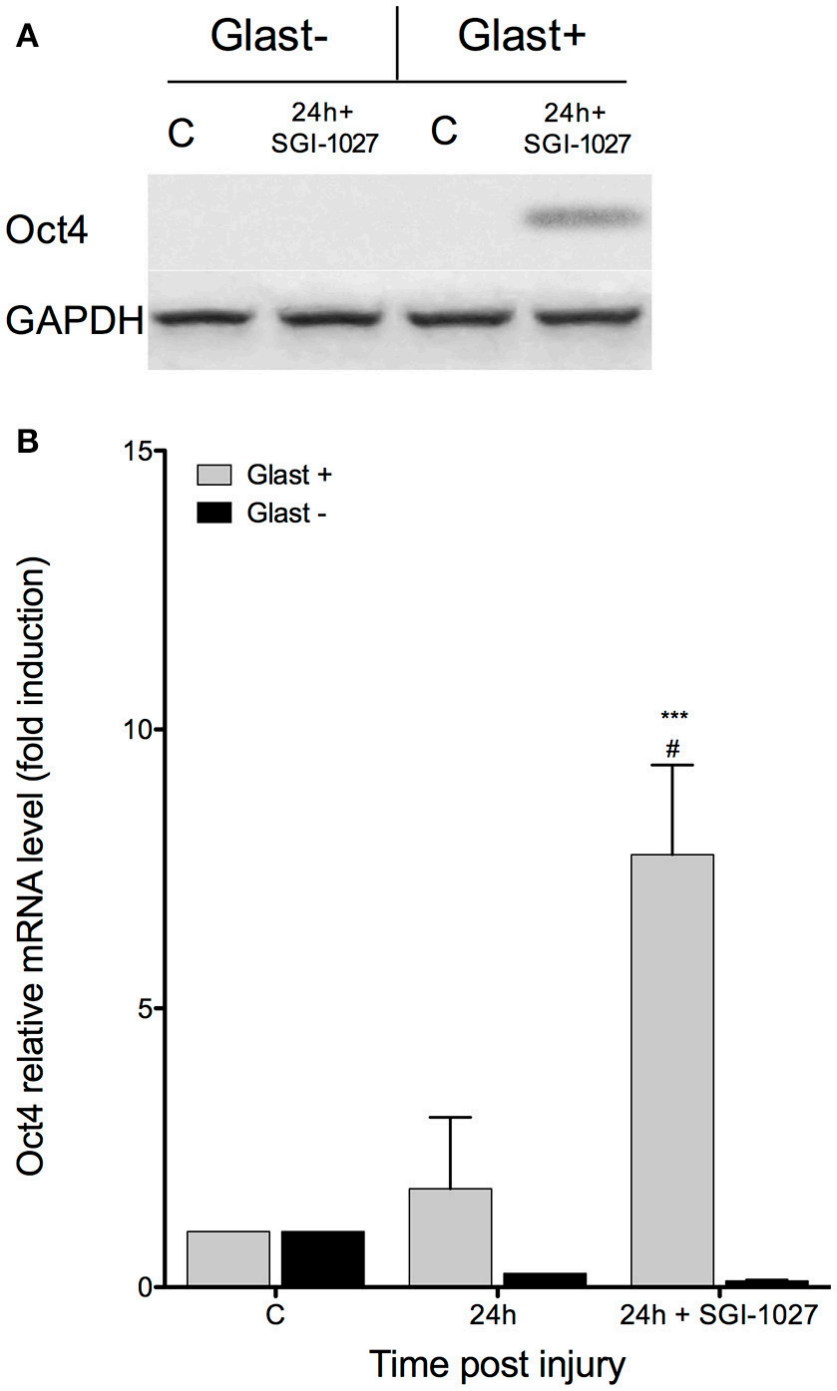
***SGI-1027 sustains Oct4 expression at 24 hpi*. (A)** RT-PCR analysis for Oct4 in MACS GLAST-positive and negative fractions of retinas treated with SGI-1027; C, intact retina as control. **(B)** qPCR quantification of Oct4 expression at 24 hpi in untreated retinas and those treated with SGI-1027. (Student's *t*-test; ^***^p < 0.001, when compared to controls; ^#^*p* < 0.001, when compared with Oct4 expression in untreated retina at 24 hpi).

To demonstrate a causal relationship between DNA methylation and *Oct4* silencing, we intravitreally administered SGI-1027, a DNA-methyltransferase inhibitor which has been shown to block and degrade DNMT1, DNMT3a, and DNMT3b (Yoo et al., 2013; Gros et al., 2015), to a group of mice (*n* = 10, 5 per condition). We evaluated the expression of *Oct4* in GLAST-positive and negative fractions of retinas injured in the presence and absence of SGI-1027. The retinas were extracted 24 h after NMDA injection, since the aforementioned pluripotency-associated marker was silenced at this time in previous experiments. Our results show that SGI-1027 allows the sustained expression of *Oct4* after retinal injury, only in the GLAST-positive fraction of retinas (Figure 6A). This increase was revealed to be statistically significant (Student's *t*-test) by qPCR analysis (*p* < 0.001, when compared to control; *p* < 0.001 when compared to damaged retinas at 24 hpi without SGI-1027 treatment; Figure 6B). These results suggest that DNA methylation could be involved in *Oct4* silencing at 24 hpi *in vivo*, and restrict this response to MG.

## Discussion

In this study, with the purpose of unveiling the critical mechanisms that impair damage- induced Müller glia dedifferentiation in mammals, we used an experimental murine model to study the kinetics and regulatory mechanisms of the expression of pluripotency-associated genes at early times after injury. We contrasted our results to thoroughly characterized data obtained in fish, and we identified, in mice, a silencing event upon *Oct4* expression that is not evident in the regenerating fish retina. We demonstrate that this event, that is restricted to Müller glia, correlates with a significant decrease in DNA methyltransferase expression and *Oct4* methylation profile. Furthermore, we suggest that intravitreous administration of a DNA-methyltransferase inhibitor refrains this silencing event *in vivo* and induces a sustained expression of *Oct4* after injury.

Seminal work has described the changes in gene expression at early time points after retinal injury in zebrafish MG, revealing a reprogramming process that drives these cells from a glial to a progenitor-like phenotype, and involves a significant increase in mRNA levels of pluripotency-associated genes (Ramachandran et al., 2010). While this particular study focused on the role of *ascl1a* and *lin28*, it also described changes in *oct4* throughout the regeneration process. This pluripotency gene is expressed in zebrafish quiescent MG and increases its levels after retinal injury; its expression is then sustained for several days, and tends to revert to basal levels a week after damage onset. The presence of a pluripotency-associated transcription factor in basal MG has been considered a reflection of its amenability to revert to a less-differentiated state and initiate retinal regeneration. Our results regarding *Oct4* expression in injured mammalian retina greatly differ from the aforementioned observations in fish, and, to our knowledge, comprise the first characterisation of changes in the expression of *Oct4* at different time points after retinal injury in mice. We have proved that excitotoxic injury rapidly induces the expression of this pluripotency-associated gene, as early as 4 hpi.

This result is relevant as *Oct4* expression in the early stages of regeneration suggests similarities among tissue regeneration and pluripotency induction, and impels the question of how further down the road of pluripotency lies the dedifferentiated MG. While no direct observations of *Oct4* effect on retinal regeneration have been reported, there is evidence of impaired fin regrowth by its knockdown at several points during regeneration in zebrafish (Christen et al., 2010). The levels of this gene are not up-regulated in regenerating tissue at levels comparable to those of iPSCs, so it might be insufficient to confer real pluripotency. However, its expression may fulfill a stoichiometric requirement that might enable cells to dedifferentiate to a multipotent state (Eminli et al., 2008; Christen et al., 2010). This possibility deserves further investigation.

Our observations contribute to establishing the dynamics of *Oct4* in the damaged mammalian retina and its striking differences from zebrafish. The demonstration that *Oct4* expression peaks shortly after retinal injury and is rapidly silenced raises the question of whether such silencing constitutes one, of many, mechanisms preventing the successful dedifferentiation of MG, and potentially cell regeneration, in mammals. From the assumption that the core idea of dedifferentiation (that is, the deletion of a genetic program and subsequent acquisition of a different one) involves a certain kind of reprogramming, we could speculate that the rapid repression of *Oct4* may reflect the fast onset of a mnemonic mechanism, potentially involved in the maintenance of glial identity.

Having observed a sudden silencing of *Oct4* after 12 hpi, we reasoned that this pluripotency-associated gene might be rapidly methylated. There is a well-documented correlation between DNA methylation and gene repression. The relative stability and heritability of DNA methylation establishes it as the main mechanism of epigenetic memory, understood as the remaining characteristics of original chromatin after reprogramming (Vierbuchen and Wernig, 2012). We measured the expression levels of enzymes responsible for the addition of methyl groups to DNA (DNMTs) and proteins associated with demethylation. Our results indicate that maintenance *Dnmt1* (Bostick et al., 2007) kept its expression levels at all times after injury, whereas we observed a significant decrease in *Dnmt3b*, that coincided with the expression peak of *Oct4*. This enzyme is traditionally considered a *de novo* methyltransferase, responsible of initiating methylation of previously unmethylated DNA (Chen et al., 2003). Orthologs to this enzyme have been identified in zebrafish, and are expressed at critical points during development of the lens and retina, particularly in the ciliary marginal zone (CMZ), where they are thought to maintain the proliferative properties of this area (Seritrakul and Gross, 2014; Takayama et al., 2014). While extensive characterization of DNMTs expression in mammalian retina is lacking, there is a report of low levels of these enzymes in MG, when compared with other cell types in the neural retina, which might account for its progenitor-like properties (Nasonkin et al., 2011).

It has been observed that *Dnmt3b* orthologs are expressed in blastema cells during fin regeneration in zebrafish, following an interesting expression pattern, i.e., they are absent before and immediately after amputation, and then their expression is induced 3 days after damage, suggesting a crucial involvement in the recovery of 5-methyl-cytosine (5 mC) levels (Takayama et al., 2014). Our results resemble these observations. The significant reduction, and further return to basal levels, in *Dnmt3b* expression that we observe may reflect the involvement of this enzyme in the recovery of methylation levels, in our case, of *Oct4* and other pluripotency-associated genes. To our knowledge, there are no previous reports regarding DNMTs in injured mammalian retina.

Accompanying *Dnmt3b* dynamics, we observed a significant increase in *Gadd45b* expression levels. This gene encodes the protein GADD45b, associated with DNA demethylation, a process that has been deemed necessary for a proper dedifferentiation. GADD45 proteins participate in repair-based DNA demethylation by recruiting the base-excision or the nucleotide-excision repair machinery (BER and NER, respectively; Jung et al., 2007; Schäfer, 2013). Interestingly, it has been reported that *Gadd45* gene expression can be induced by cellular stress (Hollander and Fornace, 2002), ultimately initiating GADD45-mediated DNA demethylation which, notably, seems to be restricted to single genes (Schmitz et al., 2009; Gavin et al., 2012; Schäfer et al., 2013), without affecting global DNA demethylation (Engel et al., 2009).

These previous results lead us to speculate about a potential correlation between *Gadd45b* expression peak and *Oct4* induction. Our findings may suggest an effect of this demethylation system on *Oct4*, which might be rapidly reversed by *Dnmt3b* return to basal levels and recovery of methylation. However, more tests are needed to fully demonstrate this notion. It is known that *Oct4* expression can be induced by *Gadd45* transfection; also, *Oct4* demethylation is inhibited by *Gadd45a* knockdown in *Xenopus* (Barreto et al., 2007), so this suggested correlation might be plausible. While data on DNA demethylation after retinal injury are still scarce, it has been reported that *Gadd45b* is induced during the transition from MG to MG-derived progenitors in injured zebrafish (Powell et al., 2013), and during MG partial dedifferentiation after glutamate stimuli in cultured mammalian MG (Reyes-Aguirre et al., 2013).

Interestingly, we also observed a significant decrease in *Tet1, Tet2*, and *Tet3*. These genes encode the homonymous proteins, which have been shown to promote demethylation by oxidizing 5 mC recruiting excision mechanisms (He et al., 2011), or preventing the proper maintenance of methylation patterns throughout cellular divisions, since they are not recognized by DNMT1 (Valinluck and Sowers, 2007). Direct evidence of Tet role in retina regeneration is still lacking. Reports in zebrafish found discrete, often non-significant changes in Tet relative mRNA levels during transition from MG to MG-derived progenitors, as well as a slight decrease 15 h post injury.

Another striking difference between our data and previously reported results in zebrafish regards the methylation state of pluripotency-associated genes. Powell et al. (2013) reported that promoters of pluripotency and regeneration-associated genes, including *Oct4*, are hypomethylated in quiescent MG. This may contribute to the progenitor-like properties of these cells (Powell et al., 2013). Surprisingly, these authors reported the same hypomethylation of the *Oct4* promoter in basal mouse MG. Our HRM results seem to contradict these findings, since we did not observe *Oct4* expression in intact retinas, nor in quiescent, MACS-separated Müller cells. The pluripotency-associated gene was only induced after retinal injury, and its methylation status in control conditions was similar to a fully methylated standard. A possible explanation for this difference might lay on the evaluated regions. Instead of focusing in *Oct4* promoter, we used specific primers for the first exon, driven by previous reports regarding the close relationship between transcriptional silencing and methylation of this region (Brenet et al., 2011).

We evaluated the effect of a novel inhibitor of DNA methyltransferases, SGI-1027, on *Oct4* expression. Notably, we found that previously tested concentrations of the inhibitor (Simó-Riudalbas et al., 2011) partially revert *Oct4* silencing at 24 hpi. SGI-1027 is a quinolone derivative, which displays inhibitory activity toward DNMT1, DNMT3A, and DNMT3B (Datta et al., 2009). It has been previously reported that regulation of DNA methylation accompanies the reprogramming of MG to progenitor cells in zebrafish, and that global inhibition of methylation by 5-dAza may activate genes associated to reprogramming, but also to proliferation, migration, and differentiation (Powell et al., 2013). These evidences comprise the only precedent of DNA methylation inhibition in injured retina, and its contribution to MG reprogramming.

Studies regarding the effect of *Oct4* expression maintenance on later stages of retina regeneration, are still needed. However, we would like to speculate that preventing the silencing of this pluripotency-associated gene at specific times after injury might improve the transition from MG to retinal progenitors, driving the process of MG-mediated retina regeneration in mammals one step further.

## Author contributions

LR and ML conceived the experiments, LR carried out the experiments, LR and ML analyzed data. Both authors were involved in writing the article and had final approval of the submitted and published versions.

### Conflict of interest statement

The authors declare that the research was conducted in the absence of any commercial or financial relationships that could be construed as a potential conflict of interest.
